# Did maize domestication and early spread mediate the population genetics of corn leafhopper?

**DOI:** 10.1111/1744-7917.12555

**Published:** 2018-01-10

**Authors:** Julio S. Bernal, Amanda M. Dávila‐Flores, Raul F. Medina, Yolanda H. Chen, Kyle E. Harrison, Kimberly A. Berrier

**Affiliations:** ^1^ Department of Entomology Texas A&M University College Station Texas USA; ^2^ Department of Plant and Soil Sciences University of Vermont Burlington Vermont USA

**Keywords:** *Dalbulus maidis*, haplotype network analysis, perennial teosinte, population genetic structuring, *Zea diploperennis*, *Zea mays mays*

## Abstract

Investigating how crop domestication and early farming mediated crop attributes, distributions, and interactions with antagonists may shed light on today's agricultural pest problems. Crop domestication generally involved artificial selection for traits desirable to early farmers, for example, increased productivity or yield, and enhanced qualities, though invariably it altered the interactions between crops and insects, and expanded the geographical ranges of crops. Thus, some studies suggest that with crop domestication and spread, insect populations on wild crop ancestors gave rise to pestiferous insect populations on crops. Here, we addressed whether the emergence of corn leafhopper (*Dalbulus maidis*) as an agricultural pest may be associated with domestication and early spread of maize (*Zea mays mays*). We used AFLP markers and mitochondrial *COI* sequences to assess population genetic structuring and haplotype relationships among corn leafhopper samples from maize and its wild relative *Zea diploperennis* from multiple locations in Mexico and Argentina. We uncovered seven corn leafhopper haplotypes contained within two haplogroups, one haplogroup containing haplotypes associated with maize and the other containing haplotypes associated with *Z. diploperennis* in a mountainous habitat. Within the first haplogroup, one haplotype was predominant across Mexican locations, and another across Argentinean locations; both were considered pestiferous. We suggested that the divergence times of the maize‐associated haplogroup and of the “pestiferous” haplotypes are correlated with the chronology of maize spread following its domestication. Overall, our results support a hypothesis positing that maize domestication favored corn leafhopper genotypes preadapted for exploiting maize so that they became pestiferous, and that with the geographical expansion of maize farming, corn leafhopper colonized *Z. diploperennis*, a host exclusive to secluded habitats that serves as a refuge for archaic corn leafhopper genotypic diversity. Broadly, our results help explain the extents to which crop domestication and early spread may have mediated the emergence of today's agricultural pests.

## Introduction

The study of crop domestication and agricultural evolution is relevant to understanding contemporary agriculture and can inform the design of agricultural systems to sustainably feed and clothe society into the future. However, while a substantial amount of research addresses fundamental agronomic questions concerning domestication and agricultural evolution, such as their environmental contexts (e.g., Araus *et al*., 2003, 2007, 2014), less research specifically addresses the relevance of crop domestication and early spread and improvement to contemporary pest management (Chen *et al*., 2015; Chen, 2016). Indeed, while much is known concerning the chronology and genetics of domestication and spread of important crops, for example, maize (*Zea mays mays* L.) (Staller *et al*., 2006; Kato *et al*., 2009; Staller, 2010; Hufford *et al*., 2012; Blake, 2015), comparatively little is known about how today's pest assemblages may have been shaped partly by agricultural, ecological and evolutionary processes over the last ∼10K years. For example, weakening of herbivore defenses by domestication may have facilitated host shifts to poorly defended and abundant crop plants, while promoting reproductive isolation of herbivore populations left behind on ancestral host plants (Medina, 2012, 2017; Medina *et al*., 2012; Chen, 2016; Forbes *et al*., 2017). Similarly, crop domestication may have altered trophic interactions among crop plants, herbivores, and herbivore antagonists, such as parasitoids, so that herbivores are more abundant on crop plants compared to crop wild ancestors (Macfadyen & Bohan, 2010). For example, common‐garden studies showed that the abundances of important pests of sunflower and maize were higher in crops compared to crop wild ancestors, partly due to enhanced performance and lower parasitism of those pests on the former compared to the latter hosts (Bernal *et al*., 2015; Chen, 2016). Overall, the evidence accumulated to date shows that crop domestication and the spread of agriculture mediated the evolution and ecology of herbivores that are presently considered pests (Medina *et al*., 2012; Chen *et al*., 2015; Chen, 2016; Medina, 2017; Forbes *et al*., 2017).

Corn leafhopper (*Dalbulus maidis* Delong & Wolcott) (Hemiptera: Cicadellidae) on maize is an ideal system for examining how crop domestication and the early spread of farming may have shaped the ecology and evolution of pests: It is a specialist on *Zea* (Poaceae), it adopted maize as a result of a host‐shift from Balsas teosinte (*Z. mays* ssp. *parviglumis* Iltis & Doebley), maize's immediate ancestor, and presently it is an important maize pest and disease vector in the American Neotropics (Nault, 1990; Medina *et al*., 2012). Prior studies uncovered clear population genetic structuring in corn leafhopper, and suggested that weaker defenses in maize compared to the crop's wild relatives may underlie the genetic structuring (Medina *et al*., 2012; Bellota *et al*., 2013; Dávila‐Flores *et al*., 2013). Specifically, those studies showed that in western Mexico corn leafhopper is structured into two genetically distinct populations: one widespread and associated with both maize and Balsas teosinte, and another secluded and associated with the geographically restricted, maize wild relative, perennial teosinte (*Z. diploperennis* Iltis, Doebley & Guzmán). Genetic structuring was hypothesized to be the outcome of a host shift, from maize to perennial teosinte, a shift facilitated by the spread of maize cultivation from Balsas teosinte's subtropical lowland habitat into perennial teosinte's highland, mesophyllous forest habitat (Medina *et al*., 2012). This hypothesis conforms with the chronology of maize domestication and spread, namely domestication began ∼9K YBP (years before present) in the lowlands of central, western Mexico, and maize cultivation was widespread in the vicinity of perennial teosinte's habitat by 7K YBP, where it intensified ∼1K YBP and declined afterwards (Kelly, 1945; Benz *et al*., 1990; Laitner‐Benz & Benz, 1994; Matsuoka *et al*., 2002; Piperno *et al*., 2007; Figueroa‐Rangel *et al*., 2010, 2012; Zizumbo‐Villareal & Colunga Garcia‐Marin, 2010). This study used AFLP marker and haplotype analyses to test that hypothesis within the relevant chronological context of maize's domestication and spread.

Haplotype analyses are relevant to this study in various ways. They can provide insight on historical processes mediating contemporary herbivorous pest distributions and host plant associations, including isolation events. Also, haplotype analyses are useful for reconstructing the evolutionary histories of populations (Ahern *et al*., 2009; Yuan *et al*., 2010; Ballman *et al*., 2011), and where populations are genetically structured, can be used to infer population ancestry on the basis of haplotype diversity and relationships (Havill *et al*., 2006). Additionally, haplotype analyses can provide insight on the origin of population genetic structuring by correlating the timing of population divergences with known isolation events (Ahern *et al*., 2009). Importantly, mitochondrial DNA (mtDNA) sequences, such as those used in this study, have been used for haplotype analyses addressing phylogenetic relationships among populations (Shufran *et al*., 2000; Anstead *et al*., 2002; Zhao *et al*., 2003; Giordano *et al*., 2005; Boykin *et al*., 2007). Recently, mtDNA and ribosomal markers were used to assess haplotypic diversity between Mexican and Argentinean samples of corn leafhopper (Palomera *et al*., 2012). Using mitochondrial cytochrome oxidase subunit I (*COI*) and the ribosomal internal transcribed spacer (*ITS2*), Palomera *et al*. (2012) did not find haplotypic variation (i.e., a single haplotype was found) among corn leafhopper samples collected from Mexico and Argentina, though their sample size was small. In this study, we used a larger and more representative sample of leafhopper specimens and the same mitrochondrial *COI* region used by Palomera *et al*. to examine corn leafhopper's recent evolutionary history and help us infer on processes underlying its emergence as a maize pest.

This study examined corn leafhopper haplotype diversity and population genetic structuring to shed light on the question of whether its evolution to an agricultural pest was associated with maize domestication and early spread. We specifically asked whether (i) maize domestication and early spread and (ii) corn leafhopper's emergence as a pest are reflected in the insect's genetic structuring and evolutionary history and diversity. In so doing, we addressed an earlier hypothesis positing that a subset of corn leafhopper genotypes were preadapted for successfully colonizing and exploiting maize before the crop's domestication (Nault, 1990; Medina *et al*., 2012). Per that hypothesis, domestication and spread of maize would have provided a novel and ecologically apparent host upon which preadapted genotypes could expand corn leafhopper's host range, distribution, and abundance. In becoming abundant and widespread upon shifting to maize, preadapted corn leafhopper genotypes would become “pestiferous” on the crop, while other genotypes remained on maize wild relatives, such as perennial teosinte. Preadaptation implies availability of standing (adaptive) genetic variation in ancestral corn leafhopper populations upon which divergent selection may act; standing variation was found to be fundamental for genetic structuring and host shifts in herbivorous insects in a recent review (Forbes *et al*., 2017). If some corn leafhopper genotypes were indeed preadapted to maize so that a shift to the crop could be rapid, we expected to find that the chronology of maize domestication and subsequent spread would be reflected in the evolutionary history and relationships of the insect's populations, if such populations exist. Specifically, the present study used corn leafhopper mtDNA sequences and AFLP markers to address whether corn leafhopper's evolutionary history, emergence as a pest, and contemporary genetic structuring and haplotype diversity are correlated with the known chronology of maize domestication and early spread. In particular, mtDNA sequences were used to infer on corn leafhopper's evolutionary history and contemporary haplotype diversity, while AFLP markers for inferring on contemporary genetic structuring. The corn leafhopper mtDNA sequences and AFLP markers were obtained from specimens collected in central, western Mexico, an area encompassing the centers of diversity of *Dalbulus* and *Zea* and of maize domestication (Nault, 1990; Buckler & Stevens, 2005), as well as on publically available DNA sequences from specimens collected from maize in Argentina (Palomera *et al*., 2012).

## Materials and methods

### Insect samples

**Figure 1:**
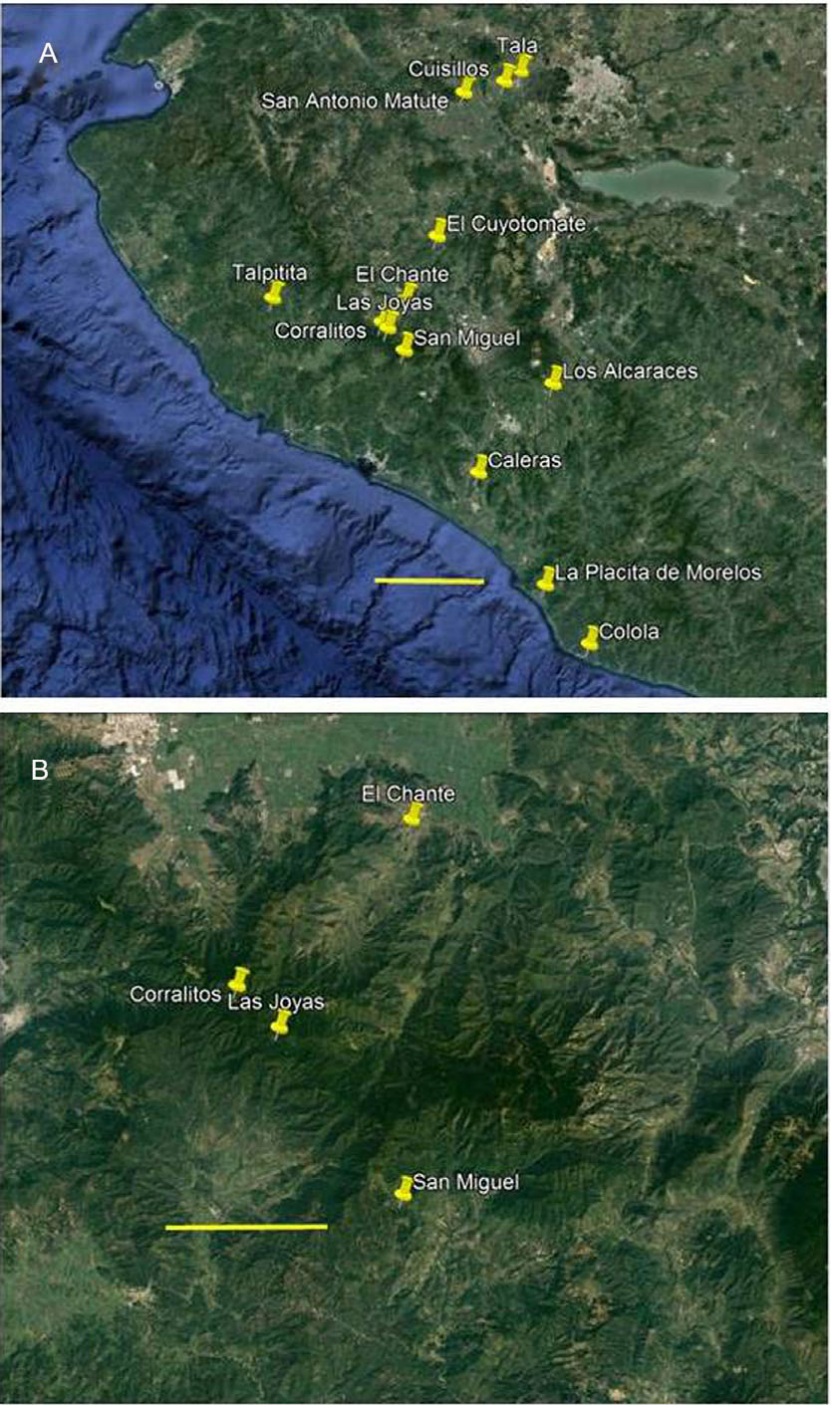
Locations in central, western Mexico where *Dalbulus maidis* samples were collected from maize (*Zea mays mays*) and/or perennial teosinte (*Zea diploperennis*). (A) All 13 locations where *D. maidis* specimens were collected. Maize is not cultivated at Las Joyas, so samples were collected exclusively from perennial teosinte at this location. Both maize and perennial teosinte grow at Corralitos and San Miguel, where samples were collected from both host plants. Maize is cultivated and perennial teosinte does not grow at the remaining locations, where all samples were collected from maize. Inset line at bottom represents 50 km. (B) Closer view of Las Joyas and nearest locations, Corralitos, San Miguel, El Chante; maize cultivation is widespread at El Chante, within 15 km of Las Joyas where only perennial teosinte grows. Maize cultivation is widespread also in the Autlán–El Grullo valley to the north of Las Joyas, as well as the Cuautitlán, Cuzalapa, and San Miguel valleys to the south. Haplotype analyses considered all 13 locations; AFLP analyses considered only Las Joyas, Corralitos, and San Miguel. Inset line at bottom represents 5 km.

Corn leafhopper adult specimens for haplotype analysis were collected from 13 sites in Jalisco, Colima, and Michoacán states, Mexico between 01 and 28 October 2011, when maize was in late reproductive stage (R4, R5) (Fig. 1A). The leafhopper specimens were collected from maize at 12 sites, and from perennial teosinte at three sites (Table 1; Fig. 1A). An earlier study showed that corn leafhoppers from maize and its ancestor Balsas teosinte formed a single, genetically discrete population (Medina *et al*., 2012). Specimens were collected by sweep‐netting within maize fields or stands of perennial teosinte, and stored in 95% EtOH. Sweep‐netting was conducted in 20–40 m row segments in maize fields or transects in perennial teosinte stands to reduce the likelihood that closely related individuals were sampled. Sample sizes per site and host plant are provided in Table 2. All three perennial teosinte sites are within the Sierra de Manantlán Biosphere Reserve (UNESCO, 2011) (Fig. 1B); these samples represented three of the four known sites where perennial teosinte occurs (the known, fourth site was sought, but the plant was not found) (Benz *et al*., 1990). Corn leafhopper specimens were collected from both perennial teosinte and maize at two sites (San Miguel, Corralitos) where perennial teosinte stands grow within ∼500 m of maize fields, while specimens were collected exclusively from perennial teosinte at one site, Las Joyas, where maize cultivation is proscribed. The Las Joyas site is located within the Reserve's “core” area, where most human activities, including maize agriculture, have been proscribed for ∼30 years (Benz *et al*., 1990; Medina *et al*., 2012). Small, scattered maize plots are ∼4 and ∼13 km distant from the Las Joyas site, at the Corralitos and San Miguel sites, respectively, while widespread maize cultivation is ∼15 km distant at the El Chante site and other areas (Fig. 1A; Table 1). Additionally, 11 publically available mtDNA sequences were included in the analyses, as described below (also, see Table 2) (Palomera *et al*., 2012).

**Table 1:** Locations in central, western Mexico where *Dalbulus maidis* samples were collected from perennial teosinte (*Zea diploperennis*) and/or maize (*Zea mays mays*). Haplotype analyses considered all locations listed; AFLP analyses considered only the first three locations (Las Joyas, Corralitos, San Miguel), which are indicated in italic type

Location	Distance (km)†	Host plants‡	Elevation (m)§	Latitude N	Longitude W
Las Joyas	–	PT	1860	19°35′32.06″	104°16′52.29″
Corralitos	4	PT, M	1810	19°36′54.62″	104°18′21.88″
San Miguel	14	PT, M	1540	19°29′59.08″	104°12′28.59″
El Chante	15	M	912	19°42′27.64″	104°12′14.20″
El Cuyotomate	48	M	1270	19°58′10.39″	104°04′01.00″
Talpitita	55	M	370	19°42′47.37″	104°47′18.33″
Caleras	78	M	80	18°59′51.07″	103°52′56.82″
Los Alcaraces	80	M	1110	19°21′48.02″	103°33′34.85″
San Antonio Matute	114	M	1255	20°33′47.58″	103°57′12.79″
Cuisillos	125	M	1280	20°36′31.86″	103°46′33.16″
Tala	132	M	1320	20°39′5.29″	103°42′26.85″
La Placita de Morelos	140	M	15	18°32′14.94″	103°35′32.36″
Colola	173	M	20	18°17′39.44″	103°24′10.98″

^†^Linear distance from Las Joyas in kilometers.

^‡^Host plants present and sampled at each location: PT = perennial teosinte, M = maize.

^§^Elevation above sea level in meters.

**Table 2:** Haplotype diversity parameters for *Dalbulus maidis* samples from central, western Mexico collected from perennial teosinte (*Zea diploperennis*) and/or maize (*Zea mays mays*). Samples include 11 available sequences from GenBank, and are listed as “Jalisco state” and “Argentina” (Palomera *et al*., 2012). In “Host plant” column, PT = Perennial teosinte (*Zea diploperennis*) and M = maize (*Zea mays mays*)

Location	Host plant	Haplotypes present (*h*)	Number of sequences (*n*)	Haplotype diversity (*Hd*)	Avg. nucleotide differences (*K*)	Nucleotide diversity (*π*)
Las Joyas	PT	B, E	18	0.1111	0.3333	0.00095
Corralitos	PT	D, E	9	0.2222	0.4444	0.00127
San Miguel	PT	C, E	6	0.3333	0.6667	0.00190
Corralitos	M	E	18	0.0000	0.0000	0.00000
San Miguel	M	E	11	0.0000	0.0000	0.00000
El Chante	M	E	12	0.0000	0.0000	0.00000
El Cuyotomate	M	E	7	0.0000	0.0000	0.00000
Talpitita	M	E	14	0.0000	0.0000	0.00000
Caleras	M	E	12	0.0000	0.0000	0.00000
Los Alcaraces	M	A, E, F	13	0.2949	0.7692	0.00220
San Antonio Matute	M	E	12	0.0000	0.0000	0.00000
Cuisillos	M	E	12	0.0000	0.0000	0.00000
Tala	M	E	18	0.0000	0.0000	0.00000
La Placita de Morelos	M	E	9	0.0000	0.0000	0.00000
Colola	M	B, E	17	0.1177	0.3529	0.00101
Jalisco state†	M	E	3	0.0000	0.0000	0.00000
Argentina	M	E, G	8	0.2500	0.2500	0.00071

^†^Includes two sequences from maize, and one from Balsas teosinte (*Zea mays parviglumis*).

Corn leafhopper adult specimens for AFLP analysis were collected with sweep net and aspirator from three sites in Jalisco state, as described above (Table 1; Fig. 1B). Specimens were collected from both maize and perennial teosinte at two sites, San Miguel (*N* = 18) and Corralitos (*N* = 20), and from perennial teosinte at one site, Las Joyas (*N* = 10). Maize and perennial teosinte grow in close proximity to each other at the San Miguel and Corralitos sites, while only perennial teosinte grows at the Las Joyas site, as noted above. The linear distances between the Las Joyas and Corralitos, and the Las Joyas and San Miguel sites are ∼4 km and ∼13 km, respectively (Table 1; Fig. 1B), while distances between the maize and perennial teosinte sampling locations within the Corralitos and San Miguel sites were ∼100 m and ∼500 m, respectively.

Except for corn leafhopper specimens from perennial teosinte, all specimens were collected from private land with permission of the respective owners. Corn leafhopper specimens from perennial teosinte were collected under permit FAUT‐0215 (Dirección General de Vida Silvestre, SEMARNAT, Mexico). As an agricultural pest, corn leafhopper is neither an endangered or protected species.

### DNA isolation

Genomic DNA for haplotype and AFLP analyses was extracted from whole, individual, adult corn leafhoppers using Qiagen DNeasy kits, following the manufacturer's protocols (Qiagen, 2002). For haplotype analyses, the average number of samples per site and host–plant combination was 13 (total = 199) corn leafhopper individuals. A total of 48 adult corn leafhoppers were used for AFLP analyses. DNA concentration and purity were measured for each specimen using a NanoDrop® spectrophotometer (NanoDrop Technologies, Wilmington, DE, USA).

### DNA amplification

For haplotype analyses, a fragment of the mitochondrial cytochrome oxidase I gene (*mtCOI*) was amplified with a primer pair specific for corn leafhopper *mtCOI* region (Palomera *et al*., 2012), dalCOI fwd (5′ TAG CTC AAC CTG GGT CGT TT), and dalCOI rev (5′ TGGTAT AGG ATT GGGTCA CCA). PCR reactions were performed in a 25 μL final volume containing 1.5 μL of DNA template (minimum 50 ng), 14 μL of 2 × Terra PCR Direct Buffer (with MgCl_2_ and dNTPs), 1.5 μL of 100 μmol/L of each primer and 0.7 μL of 1.5 U/μL Terra PCR Direct Polymerase Mix. PCRs were run with a hot start of 94 °C for 5 min, followed by 25 cycles each of 30 s at 94 °C, 60 s at 58 °C, 60 s at 72 °C, and a hold step at 4 °C.

AFLP were generated using the protocol described by Vos *et al*. (1995), with slight modifications. Restriction digestion and ligation steps were performed by adding 5.5 μL of genomic DNA to 5.5 μL of a master mix containing 1.1 μL of 10 × T4 DNA ligase buffer, 1.1 μL of 0.5 mol/L NaCL, 0.55 μL of diluted bovine serum albumin (1 mg/mL), 0.05 μL of *Mse* I, 0.05 μL of *Eco*R I, 0.03 μL of T4 DNA ligase, 1 μL of *Mse* I and 1 μL of *Eco*R I adaptors and 0.61 μL of ultrapure water (18.2 mega‐ohm/cm). The entire reaction incubated at –37 °C for 2 h for adequate digestion. Subsequently, each reaction was diluted to 1 : 18 (11 μL + 189 μL) ratio with buffer TE_thin_ (15 mmol/L Tris of pH 8.0, 0.1 mmol/L EDTA). The PCR program for preselective amplification consisted of an initial denaturing step at 95 °C for 1 min followed by 20 cycles at 95 °C for 20 s, 56 °C for 30 s, and 72 °C for 1 min, 30 s with a final extension at 75 °C for 5 min, followed by 4 °C on a thermal cycler (Life Technologies, Grand Island, NY, USA). The amplified product was diluted 20 fold using 15 nmol/L Tris‐HCL buffer (pH 8.0) containing 0.1 mmol/L EDTA. For selective PCR amplification of restriction fragments, previously prepared custom primers for recognition of *Eco*R I and *Mse* I adaptors were used (i.e., EcoRI‐ACT/MseI‐CAT; Life Technologies, Grand Island, NY, USA). Fragments were visualized using fluorescent dyes attached to the 5′ end of each *Eco*R I selective amplification primer with no modification made to the *Mse* I primer. The PCR program for the selective amplification consisted of an initial denaturing step at 95 °C for 1 min, 12 cycles of 95 °C for 20 s, 65 °C for 40 s with a lowering of 0.7 °C per cycle, 72 °C for 1 min, 30 s and a final extension at 72 °C for 7 min before storing the samples at 4 °C. For haplotype analyses, PCR products were visualized on 1% agarose gels to confirm amplification of samples and that the negative controls did not amplify. PCR products were then shipped overnight on dry ice to University of Florida Interdisciplinary Center for Biotechnology Research (ICBR) for fragment analysis.

### Sequencing and fragment analysis

AFLP fragments were separated by capillary electrophoresis by a 3130 Genetic Analyzer from Applied Biosystems (Grand Island, NY, USA) and analyzed using GeneMapper 4.0® (Applied Biosystems). Because the fluorescence of the markers can vary between runs, markers were normalized using the sum of signal method (Applied Biosystems, 2005). Markers with dye signals larger than 100 fluorescent units were considered as present. All samples were visually inspected individually to make sure that the 100 fluorescent unit threshold we chose was well above background noise. Each AFLP marker was considered a locus and assumed to have two possible alleles (i.e., 0 for band absent, 1 for band present). The fragment sizes were evaluated using 1 bp bin width in GeneMapper, and only fragments between 50 and 400 bp were analyzed.

### Analytical methods

#### Haplotype analysis

Partial *COI* sequences were aligned manually in Sequencher 4.8 (GeneCodes, Ann Harbor, USA) based on published sequences by Palomera *et al*. (2012). Consensus sequences were trimmed to 350 bp. Chromatograms were visually inspected, and only sequences with unambiguous basecalls were included in analyses. Publically available mtDNA sequences (Palomera *et al*., 2012), GenBank accession numbers JN411693–JN411695 (all three from Mexican sites) and JN411696–JN411703 (all eight from Argentinean sites), were added to our COI sequences to construct a haplotype network. Relationships between mtDNA haplotypes were examined using a parsimonious haplotype network generated at the 95% connection limit with TCS v1.21 (Clement *et al*., 2000). Statistical analyses were carried out using relevant functions within DnaSP (Librado & Rozas, 2009). Genetic diversity for each site was assessed by calculating haplotype diversity (*Hd*), average number of nucleotide differences (*K*), and nucleotide diversity (*π*). Genetic differentiation between leafhoppers collected from maize or perennial teosinte was assessed using haplotype‐based statistics, *Hs* and *Hst*, and a *χ* ^2^‐test (Nei, 1987; Hudson *et al*., 1992). Parameter estimates included sequences from our sites as well as sequences from Palomera *et al*. (2012), unless indicated otherwise.

A phylogeny was constructed with the corn leafhopper mtDNA sequences using MEGA 6 (Tamura *et al*., 2013). An outgroup sequence ancestral to *Dalbulus* [*Macrostelus quadrilineatus* Forbes (GenBank EU981884)] was included in the analyses (Dietrich *et al*., 1998). Sequences were aligned using the MUSCLE program within MEGA 6. The evolutionary history was inferred using a Maximum Likelihood method based on the Tamura–Nei model (Tamura & Nei, 1993). A consensus tree was generated using a bootstrap method with 10 000 replicates. This was obtained through a heuristic search using Neighbor‐Joining and BioNJ algorithms on a matrix of pairwise distances generated by the Maximum Composite Likelihood (MCL) approach, and then selecting the topology with superior log likelihood value. The analyses for the bootstrap consensus tree involved all haplotypes found in this study plus the outgroup sequence. Codon positions included in the analyses were 1st + 2nd + 3rd + noncoding. All positions containing gaps and missing data were eliminated. There were a total of 10 579 and 105 positions in the final dataset for the bootstrap consensus tree.

We addressed whether our inferred evolutionary history was correlated with the chronology of maize domestication and spread by applying the RelTime method in MEGA 6 (Tamura & Nei, 1993; Tamura *et al*., 2012, 2013) to construct time‐trees calibrated per the known chronology of maize spread. Specifically, the branch length for one haplotype found exclusively in Argentina (Haplotype G, see Results, Haplotype analysis) was assumed to not exceed 4000 years, equivalent to the longest plausible presence of maize in Argentina (Iriarte *et al*., 2004; Gil *et al*., 2006). The haplotype in question was not found among our Mexican samples, so we assumed that it arose in South America; moreover, we considered that corn leafhopper arrived in present‐day Argentina subsequent to earliest maize because it reproduces only on *Zea*, and the distribution of teosintes (species of *Zea* other than maize) does not extend to South America, being restricted to Mexico and Central America (Nault, 1990; Buckler & Stevens, 2005). Accordingly, we generated a series of time trees using branch‐lengths incrementally shorter than 4000 years for the haplotype in question, namely 3.5K, 3K, 2.5K, 2.0K, 1.5K, 1.0K, 0.5K, and 0.25K years. The time trees thus generated were then contrasted with the chronology of maize domestication and spread within Mexico and to Argentina.

#### AFLP analysis

AFLP data were analyzed using Bayesian cluster analyses in STRUCTURE 2.3.2 (Pritchard *et al*., 2000; Falush *et al*., 2007). The admixture model was run for 100 000 generations with a burn‐in period of 100 000 generations for 20 iterations assuming *K* = 1 through 6 populations. The *ad hoc* Δ*K* statistic was used to predict the most likely number of populations (*K*) in the data (Evanno *et al*., 2005). Nei's unbiased genetic distances among sites and within host–plant species were calculated in AFLPsurv 1.0 (Vekemans *et al*., 2002) using the methods of Lynch & Milligan (1994) to test geographic and host‐dependent population structure, respectively. An analysis of molecular variance (AMOVA) (Excoffier *et al*., 1992) and a Principal Coordinates Analysis (PCA) were performed using GenAlEx v6.4 (Peakall & Smouse, 2006) in order to compare the partition of genetic variability among populations grouped by host plant and by the location from which the samples were obtained. Tests were conducted with 9 999 random permutations of the data. Analyses were based on 31 polymorphic AFLP loci. Average corn leafhopper DNA concentration was 70.0 ± 25.9 ng/μL, and average DNA purity was 2.08 ± 0.16, per light absorbance ratio at 260/280 nm.

## Results

### Haplotype analysis

**Figure 2:**
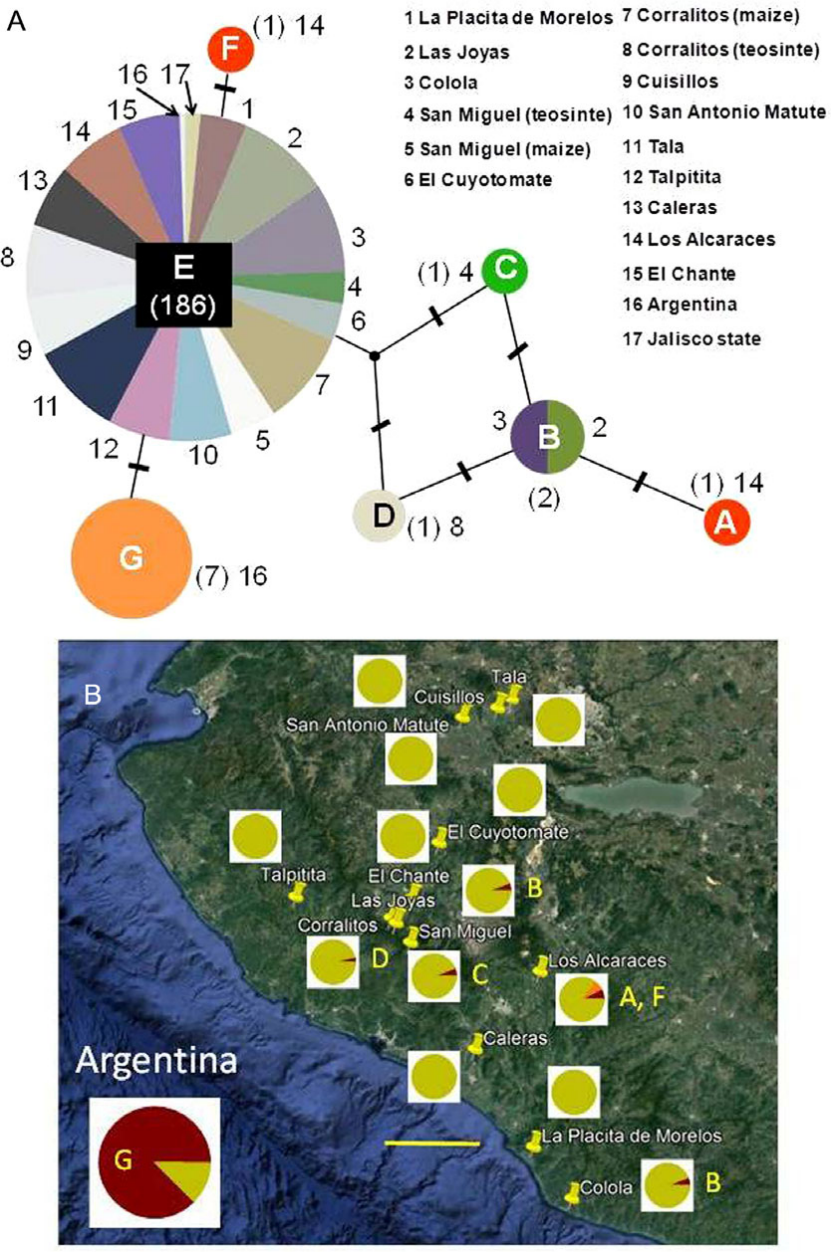
*Dalbulus maidis* haplotype network and geographical distribution. (A) Haplotype network showing relationships among seven haplotypes (A–G) found among samples collected in central, western Mexico, and Argentina; samples included 11 publically available sequences obtained from GenBank. Mexican samples were collected from maize (*Zea mays mays*) or perennial teosinte (*Z. diploperennis*); Argentinean samples were collected from maize. Upper‐case letters indicate haplotypes; numbers in parenthesis indicate numbers of corresponding sequences; numbers without parentheses indicate sampling sites listed within the figure; sequences obtained from GenBank correspond to sampling sites 16 and 17, and; tick‐marks between haplotypes indicate single base changes. Circle sizes are not proportional to the numbers of sequences corresponding to each haplotype. (B) Geographical distribution of haplotypes (A–G) in Mexico, and the composition of haplotypes at sampling sites as (inset) pie charts; Haplotype E, which is indicated by light green coloration, is ubiquitous and predominant at every sampling site. At sampling sites where haplotypes additional to Haplotype E are present they are indicated by colors other than light green, and the corresponding haplotype is indicated to the right of the pie chart. Shown at bottom left is the composition of haplotypes in Argentina, where Haplotype G is predominant and Haplotype E is subordinate. Inset line at bottom represents 50 km.

Overall, seven mitochondrial haplotypes (hereafter identified “A” through “G”) were found among the corn leafhopper specimens collected from maize and perennial teosinte in Mexico and Argentina (Fig. 2; Table 2); sequences corresponding to these haplotypes can be found in GenBank (http://www.ncbi.nlm.nih.gov/genbank/) under accession numbers KF152943–KF152948. The specimens from Mexico yielded six haplotypes, A–F, while the sequences from Argentina yielded one haplotype shared with Mexican samples, E, and an exclusive haplotype, G (Fig. 2; Table 2). Haplotype E was found in all 13 Mexican sampling sites and on both maize and perennial teosinte, as well as in both the Mexican and Argentinean samples of Palomera *et al*. (2012). Of the 199 specimens examined in this study, 186 corresponded to Haplotype E. Haplotypes A and F were unique to a single sampling site, Los Alcaraces, and each was found as single specimens collected from maize. Haplotype B was found in two localities, at Colola on maize and at Las Joyas on perennial teosinte. Haplotype C was collected only at San Miguel from perennial teosinte, and was found in a single specimen. Similarly, Haplotype D was collected only at Corralitos from perennial teosinte, and was found in a single specimen. Finally, Haplotype G was exclusive to samples from maize in Argentina, where it was found in 7 of 8 GenBank sequences (Fig. 2; Table 2). The haplotype network suggests two haplogroups, one comprising Haplotypes A–D, the other Haplotypes E–G. Overall, these results showed that: (1) Haplotype E is dominant and widespread in Mexico, on both maize and perennial teosinte, while Haplotype G is dominant and widespread on maize in Argentina; (2) Haplotypes A–D and F are rare in Mexico, and Haplotype E is infrequent in Argentina; and (3) two haplogroups may occur among Mexican and Argentinean samples of corn leafhopper.

Corn leafhopper samples from perennial teosinte were more diverse than those from maize (Tables 2 and 3). Specifically, four haplotypes, B, C, D, E, were found among 33 specimens from perennial teosinte at three sampling sites in Mexico, while five haplotypes, A, B, E, F, G, were found among 155 specimens sampled from maize at 12 sites in Mexico and 8 sites in Argentina (Table 2). Haplotype diversity (*Hd*), average nucleotide differences (*K*), and nucleotide diversity (*π*) were on average ∼4 fold greater among specimens from perennial teosinte relative to maize, indicating a significant difference in genetic diversity between samples from these host plants (*P* = 0.04) (Table 3). In particular, haplotype diversity, nucleotide differences, and nucleotide diversity were variable between modest and high among samples from perennial teosinte, while they were most frequently nil (i.e., in 11 of 14 samples) among samples collected from maize (Table 2). Overall, these results showed that corn leafhopper haplotype diversity is substantially greater on perennial teosinte than it is on maize.

**Table 3:** Haplotype and nucleotide statistics and comparison of genetic diversity between *Dalbulus maidis* samples collected from maize (*Zea mays mays*) or perennial teosinte (*Zea diploperennis*) in Mexico

Host plant	No. of sequences (*n*)	No. of haplotypes (*h*)	Haplotype diversity (*Hd*)	Avg. nucleotide differences (*K*)	Nucleotide diversity (*π*)
Maize	155	4; A, B, E, F	0.0385	0.103	0.0003
Perennial teosinte	33	4; B, C, D, E	0.1761	0.405	0.0012
		*χ^2^* = 11.419, *P* = 0.044, df = 5			

**Figure 3:**
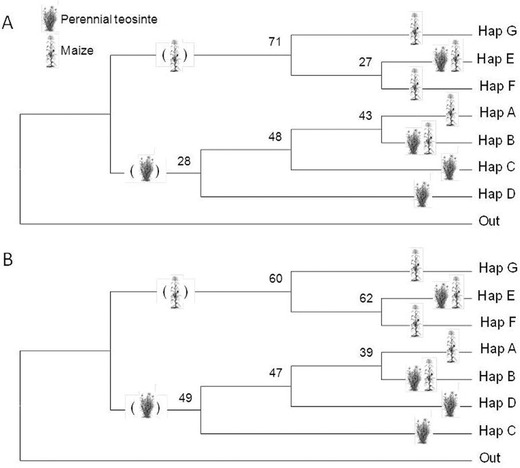
Bootstrap consensus evolutionary history of seven *Dalbulus maidis* haplotypes (A–G) inferred using (A) the Maximum Parsimony method (1000 replicates) and (B) the Maximum Likelihood method (10 000 replicates); branches corresponding to partitions reproduced in less than 50% bootstrap replicates are collapsed in both (A) and (B). External nodes are labeled with corresponding haplotype (A–G and Out; Out = *Macrosteles quadrilineatus*) and host plant (perennial teosinte, maize, or both); where maize or perennial teosinte is hypothesized hosts for clades they are inset within parentheses. The analysis involved eight nucleotide sequences. All positions containing gaps and missing data were eliminated. There were a total of 90 positions in the final dataset. Evolutionary analyses were conducted in MEGA6.

Both consensus phylogenetic trees supported the finding of two corn leafhopper haplogroups, one associated with perennial teosinte the other with maize, though the support was modest (Fig. 3). In both the maximum parsimony (Fig. 3A) and maximum likelihood trees (Fig. 3B), Haplotypes A–D and Haplotypes E–G form separate haplogroups, and the former group was positioned nearest the *Macrosteles quadrilineatus* outspecies. Haplotypes A–D were mostly associated with perennial teosinte, though Haplotype A was collected from maize as a unique specimen from a single location (Los Alcaraces) (Fig. 2; Table 2). In contrast, Haplotypes E–G were associated with maize, though Haplotype E was predominant across hosts and locations, including among specimens from perennial teosinte. Overall, these results suggested the occurrence of two corn leafhopper haplogroups, one associated with perennial teosinte, the other with maize. Notably, this result is consistent with the haplotype grouping found in the haplotype network analysis (above; Fig. 2).

Applying the RelTime method we generated a series of time trees in which the branch‐lengths of Haplotype G were set at 4000 years and shorter (not shown) (Tamura & Nei, 1993; Tamura *et al*., 2012, 2013). With branch‐length set at 4000 years we estimated that the divergence of the maize and perennial teosinte haplogroups occurred ca. 12.55K YBP; with shorter branch‐lengths, namely 3.5K, 3K, 2.5K, 2.0K, 1.5K, 1.0K, 0.5K, and 0.25K years, we obtained corresponding divergence time estimates for the maize and perennial teosinte haplogroups at ca. 11.0K, 9.5K, 8.0K, 6.4K, 4.9K, 3.4K, 1.8K, and 1.1K YBP.

### AFLP analysis

**Figure 4:**
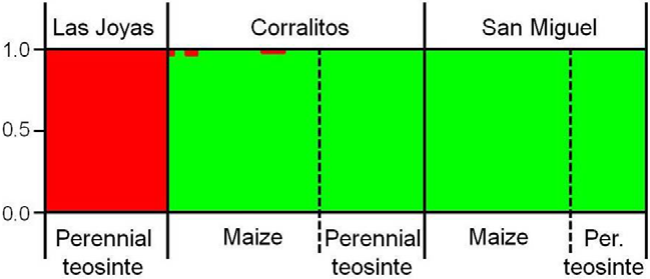
Bayesian population assignment probabilities (*y*‐axis) (in red) for *Dalbulus maidis* individuals collected from two hosts, maize (*Zea mays mays*) or perennial teosinte (*Z. diploperennis*), at three collecting sites, Las Joyas (in red), Corralitos (green), and San Miguel (green) (*x*‐axis), calculated using STRUCTURE 2.3.2. Two collection site‐associated populations are indicated. Samples from Las Joyas are genetically distinct from samples from Corralitos and San Miguel. Host‐associated structuring was not evident where both host plants coexist.

AFLP analyses revealed distinct structuring among the corn leafhopper specimens collected from perennial teosinte and maize at three collection sites, though structuring was clearly correlated to collecting site, perennial teosinte‐only site versus perennial teosinte and maize sites (respectively, Las Joyas vs. San Miguel and Corralitos), rather than host plant (maize vs. perennial teosinte) (Fig. 4). Specifically, the STRUCTURE analysis indicated that two clusters was the most likely result, amongst 1–6 clusters: at *K* = 2, all specimens were assigned to one of the two identified clusters with probability >99%, and *K* = 2 was the optimal estimation for *K* in our dataset (Fig. S1). Thus, structuring was not correlated strictly with host plant, with cluster 1‐specimens associated to perennial teosinte from Las Joyas, and cluster 2‐specimens associated to both perennial teosinte and maize at the Corralitos and San Miguel sites (Fig. 4). Importantly, further STRUCTURE analysis did not uncover substructuring within either of the two clusters (Fig. S2).

**Figure 5:**
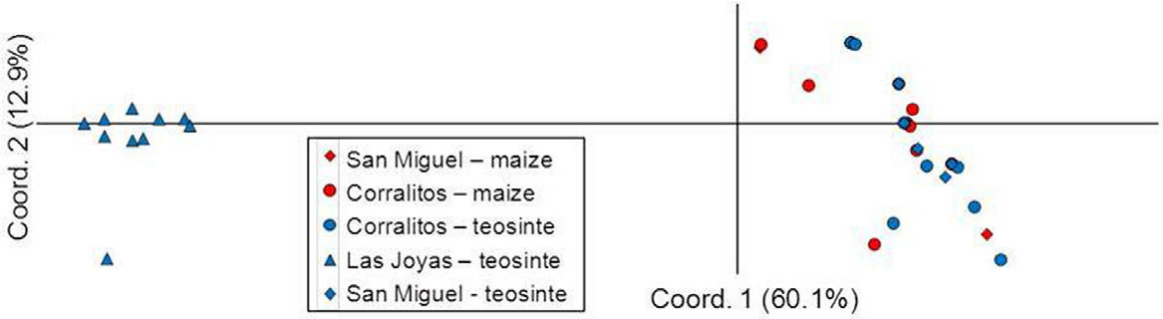
Eigenvectors of Principal coordinates 1 (*x*‐axis) and 2 (*y*‐axis) for *Dalbulus maidis* on two host plants, maize (*Zea mays mays*) and perennial teosinte (*Z. diploperennis*) from three collecting sites (Las Joyas, Corralitos, and San Miguel) (see inset text). Symbol shapes denote collecting sites, and symbol colors denote host plant species (maize = red; Perennial teosinte = blue). Principal coordinate 1 segregates populations similarly to STRUCTURE‐delimited populations (see Fig. 3).

The AMOVA results indicated that the genetic variation found among the corn leafhopper specimens was not explained by host plant, but by geographic variation, with variation within and among collection sites each accounting for 50% of total variation (Table 4). Similarly, principal components analysis revealed that Coordinate 1, which accounted for 60% of the variation, strongly segregated corn leafhoppers collected from Las Joyas (from perennial teosinte) from those collected at San Miguel and Corralitos, while Coordinate 2 accounted for 13% of the variation, but did not segregate leafhoppers on the basis of host plant or collection site (Fig. 5). Similarly, AFLPSurv *F*st statistics among collecting sites showed that genetic variation in the corn leafhopper was geographically structured, that is, *F*st between collecting sites (*F*st = 0.392, *P* < 0.001) was “very great,” while *F*st between host plants (*F*st = 0.148, *P* = 0.001) was “moderate” (*sensu* Hartl & Clark, 1997). Overall, our AFLP results confirmed those of a prior study (Medina *et al*., 2012): clear population genetic structuring in corn leafhopper in which two populations are present, one at Las Joyas on perennial teosinte and the other elsewhere on *Zea*. Importantly, this study's results showed that structuring subsides where perennial teosinte and maize grow in close proximity. Also importantly, our AFLP results were generally consistent with our mtDNA results in that they uncovered two‐group population structuring in corn leafhopper, with one population at Las Joyas (a perennial teosinte‐only site) on perennial teosinte and the other elsewhere on maize.

**Table 4:** Analysis of molecular variance (AMOVA) statistics for data corresponding to *Dalbulus maidis* samples from three collecting sites and two host plants, maize (*Zea mays mays*) and perennial teosinte (*Zea diploperennis*). Significance testing was done using 9999 permutations of the binary distance parameter δPT, an analog of *F*st in GenAlEx 6.2

Source of variation	Variance components	% variation	*P* value
Within collecting site	1.720	50%	<0.001
Among collecting sites	1.733	50%	<0.001
Among host plants	0.000	0%	0.989

## Discussion

We tested whether signals of maize domestication and early spread, and of corn leafhopper's emergence as a pest are reflected in the insect's contemporary genetic structuring and haplotype diversity. Overall, our mtDNA data uncovered seven corn leafhopper haplotypes grouped into two haplogroups: one haplogroup containing haplotypes mainly associated with perennial teosinte, the other containing haplotypes mainly associated with maize. Within the maize haplogroup was one haplotype ubiquitous and dominant at all Mexican sites, and another haplotype ubiquitous and dominant at Argentinean sites. Also, our AFLP data confirmed the two‐group, genetic structuring found in our prior study (Medina *et al*., 2012) and, importantly, the structuring was generally consistent with the mtDNA haplogrouping revealed in this study.

We addressed also an existing hypothesis positing that corn leafhopper was preadapted for successfully colonizing and exploiting maize upon its domestication (Nault, 1990; Medina *et al*., 2012). According to that hypothesis, the spread of maize agriculture provided a novel, increasingly abundant, and poorly defended host (i.e., maize), which allowed a subset of corn leafhopper genotypes to increase the insect's host range, distribution, and abundance, thus becoming pestiferous. Additionally, the hypothesis proposed that with the spread of maize agriculture beyond lowland subtropical forests, corn leafhopper colonized perennial teosinte, a novel host exclusive to highland, mesophyllous mountain forest, which presently hosts rare, plausibly archaic corn leafhopper genotypes. So, we asked whether the genetic structuring and haplotype diversity and relationships we found in corn leafhopper may on one hand be correlated with the established chronology of maize domestication and spread, and on the other hand may be reflective of the timing and context of the leafhopper's emergence as a pest.

### Are maize domestication and early spread reflected in corn leafhopper genetic structuring and haplotype diversity?

The established chronologies of maize domestication from Balsas teosinte and the crop's early spread are relevant to our study. A composite chronology indicates that maize domestication began ∼9.2K YBP (years before present) in the lowlands of central, western Mexico (Balsas River basin), rapidly spread in every direction, diversified in the central Mexican highlands, and spread from there to Central America by 6.25K YBP, Andean South America by 4.5K YBP via a western, highland route, and Atlantic South America, including present‐day Argentina by 2K–4K YBP via an eastern, lowland route (Matsuoka *et al*., 2002; Freitas *et al*., 2003; Iriarte *et al*., 2004; Gil *et al*., 2006; Vigouroux *et al*., 2008; van Etten & Hijmans, 2010; van Heerwarden *et al*., 2011). The spread of maize agriculture within present‐day Mexico was rapid: for example, maize farming was widespread in the area of domestication near the Pacific coast by 8.7K YBP, reached the Gulf of Mexico coast by 7.3K YBP, and was widespread in our study area by 7K YBP (Piperno *et al*., 2007; Pohl *et al*., 2007; Zizumbo‐Villareal & Colunga Garcia‐Marin, 2010). More specifically, maize agriculture in the vicinity of our perennial teosinte sites intensified between 1.2K and 800 YBP, after which it began declining, though archaeological and historical evidence show that maize remained common in the area prior to and at Spanish colonization, ca. 500 years ago (Kelly, 1945; Benz *et al*., 1990; Laitner‐Benz & Benz, 1994; Figueroa‐Rangel *et al*., 2010, 2012). The intensity of maize agriculture in the vicinity of our perennial teosinte sites likely tracked the dynamics of local human populations, which declined by 65%–78% with Spanish colonization in the early 1500s, rebounded to precolonization levels after 1930, and increased thereafter, though maize cultivation was proscribed at the Las Joyas site beginning in the late 1980s (Benz *et al*., 1990; Laitner‐Benz & Benz, 1994).

#### Evidence from mtDNA haplotype analysis

Our two phylogenies were consistent in suggesting that corn leafhopper haplotypes are clustered into maize and perennial teosinte haplogroups (see Fig. 3), a divergence which we hypothesize may have occurred in correlation with the chronology of maize domestication and spread. Our time‐trees calibrated with variable branch‐lengths (4K–0.25K years) for Haplotype G yielded divergence times for the maize and perennial teosinte haplogroups between ca. 12.55K and 1.1K YBP. In particular, Haplotype G branch‐lengths of 3.5K and 4K YBP yielded divergence times earlier than maize domestication beginning at 9.2K YBP, so were inconsistent with our hypothesis correlating the divergence with the chronology of maize domestication and early spread. However, branch‐lengths of 2K years and shorter yielded divergence times at ca. 6.4K YBP and later, which are consistent with our hypothesis correlating the divergence of the maize and perennial teosinte haplogroups with the chronology of maize domestication and spread. Notably, with Haplotype G branch‐lengths set at 2K years and shorter, our estimates for divergence times are particularly consistent with the timings of widespread maize farming in our study area at 7K YBP (Pohl *et al*., 2007; Zizumbo‐Villareal & Colunga Garcia‐Marin, 2010), and intensified maize farming in the vicinity of our perennial teosinte sites at 1200–800 YBP (Kelly, 1945; Benz *et al*., 1990; Laitner‐Benz & Benz, 1994; Figueroa‐Rangel *et al*., 2010, 2012).

#### Evidence from AFLP marker analysis

It is plausible that the genesis of corn leafhopper genetic structuring revealed by our AFLP data began with the declining intensity of maize agriculture in perennial teosinte habitat beginning 800 YBP (Kelly, 1945; Benz *et al*., 1990; Laitner‐Benz & Benz, 1994; Figueroa‐Rangel *et al*., 2010, 2012), and intensified beginning ∼30 years ago when maize agriculture was proscribed at our Las Joyas site following the creation of the Sierra de Manantlán Biosphere Reserve, particularly the Reserve's “core” area containing the Las Joyas site. Establishment of the core area and proscription of maize agriculture plausibly created a buffer zone partially isolating perennial teosinte habitat, so promoting reproductive isolation between the perennial teosinte population and the maize population and resulting in the genetic differentiation observed in Las Joyas (Medina, 2012, 2017; Forbes *et al*., 2017). Thus, while our AFLP results do not provide a timeline for the evolution of genetic structuring in corn leafhopper, either instance of maize farming retreat from the vicinity of perennial teosinte habitat (i.e., ∼800 or ∼30 years ago) may have promoted a localized process of habitat‐ or host‐associated differentiation at this particular location, consistent with our hypothesis derived from our haplotype analysis (above). Geographic variation in the occurrence of host‐associated differentiation has been observed in other hemipterans (Goodman *et al*., 2015; Forbes *et al*., 2017). Moreover, the durations of processes putatively beginning with either instance of maize farming retreat, whether hundreds or dozens of years, are consistent with the lengths of processes leading to genetic structuring in other insect herbivores (Winter, 1992; Claridge, 1995; Cocroft *et al*., 2008; Bennett & O'Grady, 2012; Medina, 2012, 2017; Goodman *et al*., 2015; Forbes *et al*., 2017). Thus, the results of our AFLP analysis appear to be consistent with the chronology of maize spread to and retreat from perennial teosinte habitat, as known from historical and archaeological studies (Kelly, 1945; Benz *et al*., 1990; Laitner‐Benz & Benz, 1994; Figueroa‐Rangel *et al*., 2010, 2012).

### Is corn leafhopper's emergence as a pest reflected in its genetic structuring and haplotype diversity?

#### Evidence from mtDNA haplotype analysis

The hypothesis of Nault (1990) and Medina *et al*. (2012) presumes standing genetic variation in corn leafhopper prior to maize domestication so that one or more corn leafhopper genotypes were particularly capable of colonizing and exploiting maize upon its domestication. Also, the hypothesis presumes that the genotypic diversity of maize colonists narrowed relative to ancestral diversity on Balsas teosinte, a narrowing consistent with domestication processes generally, and seemingly with the evolution of pests (Larson, 2011; Medina, 2012, 2017). Similarly, human‐driven plant range expansions, such as that of crops following their domestication, have been associated with narrowing genetic diversity in insect herbivore populations colonizing novel hosts, so that diversity is greater on the evolutionarily archaic segments of a species’ host range compared to that on modern, recently colonized hosts (Oliver, 2006; Alvarez *et al*., 2007). Thus, adoption of maize by corn leafhopper presumes a narrowing of its genetic diversity, which is consistent with our results showing that haplotype diversity and frequency of unique haplotypes were greater among samples from perennial teosinte than from maize. The greater haplotype diversity on perennial teosinte compared to maize suggests also that the diversity on the teosinte may reflect the genotypic diversity present during the early spread of maize agriculture, when the crop reached the teosinte's highland habitat. Relevant to this interpretation, Medina *et al*. (2012) suggested that perennial teosinte in its mountainous habitat at Las Joyas may act as a refuge for ancestral corn leafhopper genotypic diversity. Maize agriculture intensified and declined during the last 1200 years in the vicinity of our Las Joyas site, and, notably, maize agriculture was proscribed at that site ∼30 years ago, as noted above, which may have created a refuge where archaic genotypes (e.g., Haplotypes B–D) now persist on perennial teosinte (see Medina *et al*., 2012). Thus, both our haplotype phylogenies and network suggest that the perennial teosinte haplogroup represents genotypic diversity of ancestral corn leafhoppers on Balsas teosinte, and of early maize colonists, while the maize haplogroup represents diversity associated with maize and maize agriculture.

The marked dominances of Haplotypes E in Mexico and G in Argentina are also consistent with a narrowing of corn leafhopper genotypic diversity with its adoption of maize and the spread of maize agriculture. Because of Haplotype E's clear predominance and ubiquity in Mexico (93% of all specimens, 100% of all collection sites), we believe that it represents corn leafhopper genotypes especially successful at exploiting maize, and suggest that it may be considered “pestiferous.” Similarly, Haplotype G of the maize haplogroup is predominant and ubiquitous in Argentina, and we suggest that it is pestiferous in Argentina. The timing of emergence of Haplotype E may be gleaned in our time‐trees. In those time‐trees, the emergence (branch‐length) of Haplotype E (see Fig. 3) may have occurred ca. 1950 years ago, if branch‐length for Haplotype G is set at 4K years, an implausible scenario, as explained above. However, if Haplotype G's branch‐length is set at the seemingly more plausible lengths of 2K years and shorter (see above), then emergence of Haplotype E may have occurred as early as 950 YBP to as late as 75 years ago. These more recent emergence times for Haplotype E, the pestiferous haplotype in Mexico, seem plausible given their broad coevality with protracted, local periods of human population growth and associated agricultural intensification (Alba, 1981; McCaa, 2000; Blake, 2015).

#### Evidence from AFLP marker analysis

The hypothesis underlying our study implies that with the spread of maize agriculture corn leafhopper successfully colonized novel hosts beyond the lowland habitat shared with its ancestral host Balsas teosinte. Thus, corn leafhopper colonized highland *Zea* taxa, such as perennial teosinte and *Zea perennis* (Hitchcock) Reeves & Mangelsdorf in the western Mexican highlands, and Chalco teosinte [*Z. mays* L. ssp. *mexicana* (Schrader) Iltis] in the central Mexican highlands, which presently are known hosts (Triplehorn & Nault, 1985; Nault, 1990). In this scenario, genetic structuring among corn leafhopper populations is especially plausible in association with perennial teosinte and *Z. perennis*, given these host's putatively stronger defenses against corn leafhopper and isolated, mountainous habitats (Nault, 1990; Buckler & Stevens, 2005; Bellota *et al*., 2013; Dávila‐Flores *et al*., 2013); indeed, both of those teosintes are known exclusively from single, isolated mountain ranges in western Mexico. Thus, corn leafhopper genetic structuring was considered likely in the context of our AFLP studies (including Medina *et al*., 2012), and our present results revealed clear genetic structuring between locations, so refining the results of our earlier study (Medina *et al*., 2012). Notably, in our earlier study we did not find structuring among samples from maize from locations separated by hundreds of kilometers and geographical barriers (*F*st ≈ 0), though we found clear structuring between samples from maize and perennial teosinte separated by <15 km, as we found in this study. Our results in this and our prior study (Medina *et al*., 2012) confirm that spatial separation *per se* does not mediate genetic structuring in corn leafhopper, and suggest that host plant and habitat differences along with evolutionary histories are important mediators of genetic structuring, with maize being a poorly defended host, growing for numerous (100s–1000s) generations in typically ephemeral, highly disturbed (agricultural) habitats, and perennial teosinte a better‐defended host growing in a comparatively stable, unperturbed forest habitat.

Generally, the results of our AFLP analyses were consistent with those of our haplotype analyses in evidencing a widespread and dominant corn leafhopper population on maize and another population at the Las Joyas site on perennial teosinte. Similar to our haplotype analyses, the Las Joyas/perennial teosinte‐associated population may be considered “wild” or “nonpestiferous,” while the dominant maize‐associated population may be considered pestiferous, and its emergence correlated with agricultural intensification.

### What maintains genetic structuring in corn leafhopper populations?

Importantly, while this study's results pointed to host plant and habitat differences as likely mechanisms maintaining genetic structuring among corn leafhopper populations, alternative mechanisms are plausible, such as isolation by distance and immigrant inviability. The results of prior studies, however, do not support a role for isolation by distance because corn leafhopper dispersal and gene flow are uninterrupted across hundreds of kilometers, while clear structuring is evident at much shorter distances, such as between our Las Joyas perennial teosinte site and nearest maize (∼4 km, at Corralitos, see Table 1), and widespread maize cultivation (∼15 km, at El Chante) (Oliveira *et al*., 2007; Medina *et al*., 2012). Similarly, a prior study is not supportive of immigrant inviability as an important mediator of genetic structuring in corn leafhopper, given that while wild (nonpestiferous) females dispersing to maize from perennial teosinte were found to potentially gain a small fitness advantage over local females, pestiferous females dispersing from maize to perennial teosinte did not suffer any fitness disadvantage compared to local, wild females (Ramirez‐Romero *et al*., 2017). Thus, differences in host plant defenses and habitat persistency remain as plausible, nonexclusive variables maintaining genetic structuring among corn leafhopper populations that should be addressed in future studies.

## Conclusion

Our mtDNA analyses revealed the existence of seven haplotypes among corn leafhopper samples from 24 sites and two host plants in Mexico and Argentina. Our analyses consistently separated a maize‐associated haplogroup (Haplotypes E–G), from a perennial teosinte‐associated haplogroup (Haplotypes A–D). Among the maize haplogroup, two haplotypes stood out for their ubiquity and frequency, E from Mexico and G from Argentina, which may be considered pestiferous. The haplotypes of the perennial teosinte haplogroup may correspond to archaic genotypic diversity representative of the diversity when maize agriculture initially expanded and corn leafhopper colonized perennial teosinte. Additionally, our results showed that haplotypic diversity is lower on maize compared to perennial teosinte, coincident with the loss of genetic diversity following adoption of a novel host, and consistent with the marked dominances of Haplotypes E and G on maize, and hinting at the processes underlying the emergence of pestiferous insect populations (i.e., agricultural pests) from populations specialized on wild ancestors. Our AFLP analysis confirmed previously documented population genetic structuring in corn leafhopper, and pointed to greater habitat persistency and stronger plant defenses in perennial teosinte compared to maize as variables underlying such structuring. Overall, our results highlighted the extent that host plant and geographic range expansions may have mediated corn leafhopper's evolution as an agricultural pest in particular, and they hinted at the roles that human activities such as crop domestication and farming expansion may have played in the emergence of agricultural pests generally.

## Disclosure

The authors report no conflict of interest.

## Supporting information


**Fig. S1**. Output from STRUCTURE Harvestor web‐based program. The *x*‐axis represents the estimated number of populations (*K*) and the *y*‐axis represents the change in *K* calculated as mean (|*L*"(*K*)|)/sd(*L*(*K*)) where *L*(*K*) describes the likelihood that a given *K* is the correct number of populations represented in the data set; the spike in the trend line indicates that the optimal *K* = 2.

**Fig. S2**. STRUCTURE 2.3.4 output graphic for (A) all samples excluding the teosinte‐associated insects from Las Joyas, and (B) teosinte‐associated insects from Las Joyas only. Each column represents an individual. The colors represent two possible populations and the probability (*Y*‐axis) that a given individual belongs to one population or another. In (A), individuals have unequal probabilities of belonging to one population or another but are not structured by host‐associations or geography; in (B), individuals have equal probabilities of belonging to one population or another.
