# Supplementary figures and images for: Did maize domestication and early spread mediate the population genetics of corn leafhopper?
Source: Insect Sci. 2018 Jan 10;26(3):569–86. doi: 10.1111/1744-7917.12555 (PMC7379674; doi:10.1111/1744-7917.12555)

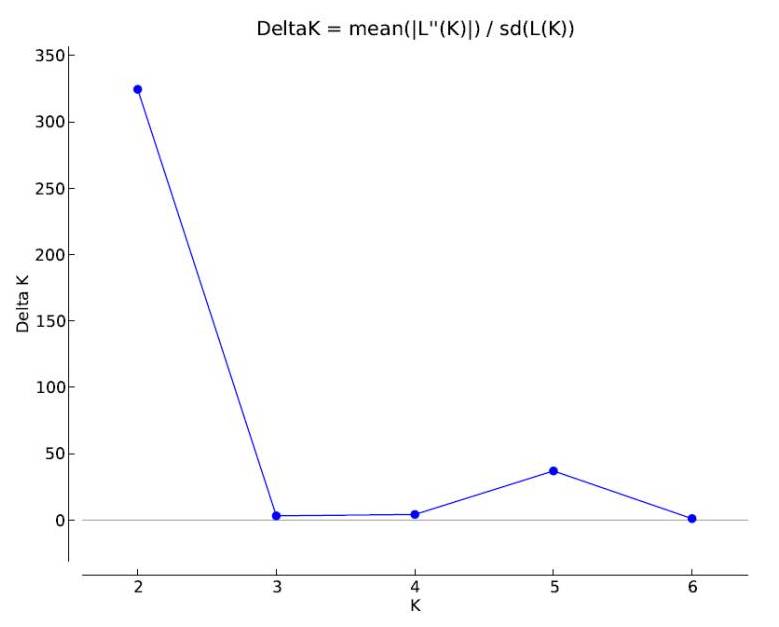

Supplement: Supplementary file 1 — Fig. S1. Output from STRUCTURE Harvestor web‐based program. The x‐axis represents the estimated number of populations (K) and the y‐axis represents the change in K calculated as mean (|L"(K)|)/sd(L(K)) where L(K) describes the likelihood that a given K is the correct number of populations represented in the data set; the spike in the trend line indicates that the optimal K = 2. [file INS-26-569-s001.jpg]

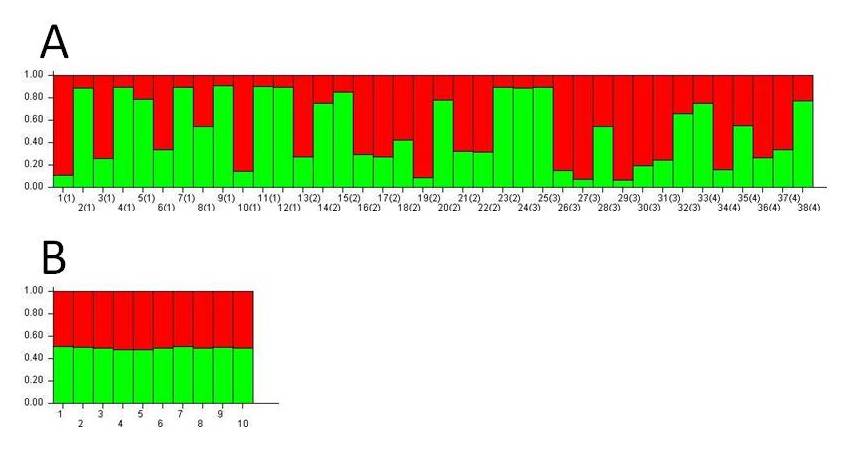

Supplement: Supplementary file 2 — Fig. S2. STRUCTURE 2.3.4 output graphic for (A) all samples excluding the teosinte‐associated insects from Las Joyas, and (B) teosinte‐associated insects from Las Joyas only. Each column represents an individual. The colors represent two possible populations and the probability (Y‐axis) that a given individual belongs to one population or another. In (A), individuals have unequal probabilities of belonging to one population or another but are not structured by host‐associations or geography; in (B), individuals have equal probabilities of belonging to one population or another. [file INS-26-569-s002.jpg]
